# County-level residential segregation and sedentary behavior in US adults

**DOI:** 10.1186/s44167-025-00084-w

**Published:** 2025-09-02

**Authors:** Mohammad Moniruzzaman, Yangyang Deng, Breanna Rogers, Sheikh Mohammed Shariful Islam, Kelly K. Jones, Pedro F. Saint-Maurice, Shreya Patel, David Berrigan, Charles E. Matthews, Kosuke Tamura

**Affiliations:** 1 Socio-Spatial Determinants of Health (SSDH) Laboratory, Population and Community Health Sciences Branch, Division of Intramural Research, National Institute on Minority Health and Health Disparities, National Institutes of Health, Bethesda, MD USA; 2 Institute for Physical Activity and Nutrition, Deakin University, Burwood, VIC Australia; 3 Neighborhoods and Health Laboratory, Population and Community Health Sciences Branch, Division of Intramural Research, National Institute on Minority Health and Health Disparities, National Institutes of Health, Bethesda, MD USA; 4 Metabolic Epidemiology Branch, Division of Cancer Epidemiology and Genetics, National Cancer Institute, National Institutes of Health, Rockville, MD USA; 5 Epidemiology and Biostatistics Department, Dornsife School of Public Health, Drexel University, Philadelphia, PA USA; 6 Behavioral Research Program, Division of Cancer Control and Population Sciences, National Cancer Institute, National Institutes of Health, Rockville, MD USA; 7 Champalimaud Foundation, Lisbon, Portugal

**Keywords:** Segregation, Isolation index, Sedentary behavior, Physical inactivity, US adults

## Abstract

**Background:**

Excessive sedentary behavior (SB) is highly prevalent among adults in the United States (US). From a socio-ecological perspective, residential segregation may affect SB; however, this remains understudied. Thus, we aimed to examine associations between county-level segregation and sedentary time in a nationwide sample of US adults.

**Methods:**

For this cross-sectional study, we analyzed data from 2,637 US adults aged 20–75 years (mean age [45.1 years], female [50.6%]) from the population-based AmeriSpeak panel in 2019. Participants completed the Activities Completed over Time in 24-hours (ACT24) previous-day recall, which is a validated population-level measure of total daily SB (hours/day). Residential segregation was expressed as the isolation index at the county level for non-Hispanic (NH) Black and Hispanic adults (vs. all other racial and/or ethnic groups). Isolation index ranges from 0 to 1, with a higher value indicating higher segregation. We used survey-weighted linear regression models to examine the relationships of race and/or ethnicity specific isolation index with total daily SB, adjusting for covariates. Models were also stratified by sex.

**Results:**

US adults reported a mean of 9.5 h/day of sedentary time, with NH Black and Hispanic adults reporting 9.8 and 8.9 h/day, respectively. NH Black segregation was not related to sedentary time (β = -0.30 [-2.53, 1.94], *p* = 0.790). Hispanic segregation also showed no relationship (β = 0.32, [-1.64, 2.28], *p* = 0.743). Moreover, sex-stratified analyses showed null associations.

**Conclusions:**

We found no association between residential segregation and SB among NH Black and Hispanic adults, and these associations did not vary by sex. Future studies should aim to replicate this study with larger samples of underrepresented minority populations.

**Supplementary Information:**

The online version contains supplementary material available at 10.1186/s44167-025-00084-w.

## Introduction

Sedentary behavior (SB), commonly assessed by time spent sitting on a typical day, has become one of the critical public health issues due to its increasing trend [1–4] and the adverse clinical consequences associated with increased SB. Recent evidence has shown that SB was associated with all-cause and cardiovascular disease mortality, cancer, and diabetes among US adults [5–16]. A recent nationwide survey showed that US adults (aged 20–75 years) spent an average of 9.5 h/day in SB [17] suggesting that many adults may be at increased risk for adverse health outcomes due to excessive SB. The National Health and Nutrition Examination Survey assessing self-reported SB among US adults indicated that time spent in SB may have increased from 5.7 to 6.4 h/day between 2007 and 2016 [1], but this increase did not appear to be sustained into 2018 [4]. Reducing time spent in SB could substantially improve health outcomes. Although research on SB has substantially increased over the last decade, greater emphasis is needed on understanding the determinants of SB [18]. Several potential determinants were identified in previous systematic reviews and meta-analyses, most of which were related to individual-level sociodemographic (e.g., age, sex, race, education, income), health-related (e.g., obesity, mood, general and mental health status), and social and physical environmental factors (e.g., social cohesion, transit density, residential location, workstation) [18–20]. However, the relationship between structural factors, such as residential segregation, and SB has remained understudied.

Residential segregation, the physical separation of racial and/or ethnic groups into different geopolitical boundaries (e.g., county, census tract), is one of the fundamental determinants of health [21] and has been shown to lead to disparities in cardiovascular disease risk factors by racial and/or ethnic groups and sex [22, 23]. Notably, residential segregation may plausibly affect behavioral mediators, such as SB, which may lead to adverse health outcomes [24, 25]. Residential segregation may correlate with built environment features [26, 27], thereby contributing to SB. Previous studies have shown that county-level built environment characteristics are associated with residents’ walking and motorized travel behavior [28, 29]. For example, one study demonstrated that county-level built environment features such as smaller block sizes (better street connectivity), higher population, and employment densities were significantly related to more walking [28]. In another study, built environment factors (average block size and employment density) at the county level were positively related to vehicle miles traveled [29]. These findings suggest that macro-level built environments can shape daily mobility patterns, including walking and motorized travel behavior, which in turn may influence SB. For instance, driving or sitting in automobiles is an important contributor to SB [30]. Moreover, county-level residential segregation was associated with higher body mass index and a greater likelihood of obesity [31, 32], both of which are known correlates of SB [18, 19], suggesting another possible pathway through which segregation may influence SB. Additionally, residential segregation may lead to increased stress and experiences of discrimination, which can reduce individuals’ interest in or prevent them from engaging in physical activity, ultimately contributing to a more sedentary lifestyle [25].

Existing research to date examining the relationship between residential segregation and SB is limited. Only one study—using the Coronary Artery Risk Development in Young Adults (CARDIA) cohort—investigated cross-sectional and longitudinal associations of neighborhood segregation with accelerometer-derived sedentary times, reporting null associations [33]. Further efforts to understand how residential segregation may be associated with SB could help explain variation in sedentary time. Additionally, examining the specific life domains in which SB occurs (e.g., work, transport, leisure, and household) may offer valuable insight for formulating targeted strategies to reduce sedentary time. However, this area remains understudied [34]. Furthermore, time spent in SB varies by sex and racial and/or ethnic groups. For example, in a recent US study, males reported more sedentary time than females (9.9 vs. 9.1 h/day). Hispanic adults reported the least (8.9 h/day) sedentary time, whereas NH White adults (9.6 h/day) and NH Black adutls (9.8 h/day) were similarly sedentary [17]. However, the reasons for such observed associations and racial and/or ethnic differences in sedentary time were not examined.

Therefore, the present study aimed to examine whether county-level residential segregation is associated with sedentary time in the nationally representative population of US adults and whether these relationships are varied by sex and specific life domains. We hypothesized that participants living in highly segregated counties would be more likely to spend more time in sedentary behaviors.

## Methods

### Study participants and design

Data from participants of the AmeriSpeak panel, a probability-based survey weighted to represent the US population (aged 20–75 years), was analyzed. The weighted analytical sample from the AmeriSpeak panel demonstrated a demographic distribution (e.g., age, sex, education, race/ethnicity, and household income) comparable to that of US adults in the Current Population Survey, reflecting the representativeness that can be obtained when using the AmeriSpeak sample [35]. Detailed methods for participant enrollment in the AmeriSpeak panel have been previously described [17, 36, 37]. The National Opinion Research Center (NORC) at the University of Chicago collected data for this study from October to November 2019.

Initially, a target time period (e.g., 7 days) was identified, and within that period, one day was selected at random. Participants were then sent electronic invitations on the randomly selected day of the week to complete a brief online survey and a recall of the previous day. Subsequently, those who completed the first recall were sent another invitation 1 to 2 weeks later to complete a second recall, again on a randomly selected day. Recalls were restricted to completion only on the targeted recall day [17]. Of 15,153 AmeriSpeak panelists invited, 2,877 completed the short survey and at least one valid recall using a previous-day recall tool that captured activities completed over time in 24 h (ACT24). From 4,575 total recalls (*n* = 2,838 first recalls, *n* = 1,737 s recalls), we excluded 408 recalls with more than 1 h per day of unknown time (*n* = 293 recalls), ≥ 2 h per day of overlapping time (*n* = 91 recalls) and recalls with 0 sedentary hours (*n* = 24). Further, we excluded 6 participants with missing values in the isolation index. After exclusions, the final analytical sample included 2,637 participants with at least one valid recall (total recalls 4,161; *n* = 2,475 first recalls, *n* = 1,686 second recalls). If participants had two valid recalls, each recall was treated as an independent observation in the analysis.

### Development of survey sample weights

The AmeriSpeak Panel enrollment employed a two-stage sampling design. Initially, the US was divided into 136 strata, such as metropolitan statistical areas and counties [36, 37]. For analysis, the 136 strata were consolidated into 47 virtual strata, each further divided into 2 to 113 virtual primary sampling units. A final panel base sampling weight was calculated for each AmeriSpeak panelist. Further, for each recall, study-specific sampling weights were calculated, adjusting for selection probabilities from the panel, non-response rate in the current study, and population coverage. The final weights were additionally adjusted to the external population totals with respect to age, sex, education, race and/or ethnicity, housing tenure, telephone status, and Census Division derived from the Current Population Survey [36, 37]. Additionally, study-specific sampling weights were calculated separately for each recall day of the week and then normalized so that each day contributed equally. This sampling method allowed us to obtain a representative sample of US adults and estimate SB at the population level [17, 35].

### Outcome

SB was assessed using the ACT24 previous-day recall instrument [17, 37–39]. ACT24 is a validated tool for estimating sedentary time at the population level compared to activPAL [38, 40]. In a study of 932 adults aged 50–74 years, ACT24 accurately estimated mean sedentary time within 1% of activPAL (9.9 vs. 9.8 h/day) [38]. Similarly, in another study of 47 adults aged 20–73 years, ACT24 was also accurate in estimating sedentary time compared with activPAL (9.1 vs. 9.3 h/day), with a non-significant mean difference of 0.17 h/day. The correlation between these measures was moderate (Spearman rho = 0.61) [40]. To complete an online recall using a smartphone, tablet or computer, participants were asked to report their activities completed on the previous day, spanning from midnight to midnight, from a list of over 170 individual activities organized in 14 major categories and six life domains (work, transport, leisure, personal, household, and other activities) [17, 37]. This included reporting time spent in bed/sleeping, engaging in physical activities, and SB. Once participants selected an activity, they answered a series of follow-up questions to determine the duration of each activity, body position, and other specific details. The reported activities were then linked to the Compendium of Physical Activities [41], and data were scored to calculate energy expenditure using metabolic equivalent (MET) values assigned to each reported activity. SB was defined as those involving sitting/reclining and little energy expenditure (typically ≤ 1.5 METs) outside of time in-bed for the primary sleep period, or during the waking day [17, 37]. Total daily time spent in SB was expressed in hours/day. Furthermore, time spent in SB across specific life domains (work, transport, leisure, personal, household, and other activities) was categorized according to ACT24 major category labels, following a classification consistent with the lexicon of the American Time-use Survey [42].

### Exposure

$$\:Isolation\:Index=\:{\sum\:}_{i=1}^{n}\left[\frac{{b}_{i}}{B}\right]*\left[\frac{{b}_{i}}{{t}_{i}}\right]$$

where$\:{\:b}_{i}$ is the number of residents who are NH Black/Hispanic individuals in the census tract $\:i$, $\:{t}_{i}$ is the total population in the tract $\:i$, and $\:B$ is the number of NH Black/Hispanic participants in the county. We quantified residential segregation using the isolation index at the county level for two racial and/or ethnic groups: NH Black and Hispanic participants. Home addresses were used to identify residences within counties. The isolation index for a particular minority group has been used to estimate the extent to which members of the minority group may be exposed only to one another, rather than to members of other racial and/or ethnic groups [43]. It also reflects the broader reality of living in segregated areal units, which are often linked to limited access to activity-friendly environments due to the underinvestment of resources needed for these facilities [21, 25]. The isolation index ranges from 0 to 1. A higher value indicates a higher probability of contact among members of the same minority group (high segregation) and, thus, a lower probability of contact with different racial and/or ethnic groups. In the current study, while calculating the NH Black isolation index, we used all other racial and/or ethnic groups (including NH White, Hispanic, Asian, and others) as the reference. Similarly, for the Hispanic isolation index, all other racial and/or ethnic groups (such as NH White, NH Black, Asian, and others) were used as the reference group. For sensitivity analysis purpose, we calculated the NH Black and Hispanic isolation indices using NH White adults as the reference group.

### Covariates

Individual level covariates included age (years), sex (male/female), marital status (married/unmarried), education (high school or less, some college/associate degree, bachelor’s degree, or graduate degree), and occupation (working for pay, not working—looking/laid off, not working—other, retired, and disabled) and body mass index (BMI). BMI was calculated by dividing participants’ self-reported body weight in kilograms by their height in meters squared and categorized into < 25.0, 25.0–29.9, and ≥ 30. Additionally, we included two areal-level covariates, county-level poverty and census regions (Northeast, Midwest, South, and West). County-level poverty was quantified using the American Community Survey five-year estimates (2016–2020) as the percentage of households living below the poverty line within each county, with a higher quartile indicating higher poverty levels.

### Statistical analysis

For continuous variables, we calculated the weighted means and standard errors (SEs). For categorical variables, we calculated the actual (i.e., unweighted) number of participants and the weighted population percentages in each category. We performed weighted multivariable linear regression to assess the relationship between county-level segregation quantified by isolation index and sedentary time using SAS Proc SURVEYREG with survey weighting. Both segregation index and sedentary time were modeled as continuous variables and adjusted for potential covariates into two models. Model 1 was adjusted for age and sex. Model 2 was additionally adjusted for marital status, educational attainment, occupational status, body mass index category, county-level poverty, and census regions. We replicated the same regression models (model 1 and model 2) stratified by sex to explore whether results vary by sex. Furthermore, we performed the same regression models to assess the relationship between county-level segregation and domain-specific sedentary time. We computed β-coefficients and the corresponding 95% confidence interval (CIs) for all regression analyses. We declared P less than the 2-sided significance level of 0.05 to be significant for all analyses. Statistical analyses were performed using SAS software, version 9.4 (SAS Institute, Cary, NC).

## Results

### Characteristics of the participants

Table 1 shows the characteristics of the participants. Mean age was 45.1 years (SE ± 0.51), and 50.6% were females. Approximately two-thirds (63.6%) identified as NH White adults, followed by 17.0% Hispanic adults, 11.0% NH Black adults, and 3.6% Asian adults, and about half of the participants (51.5%) were married. Around one-third (34.6%) had a high school education or less, 41.2% reported a household income of <$50,000, and more than two-thirds (67.2%) were currently working for pay. The mean BMI was 29.3 kg·m^− 2^, and 37.4% reported a BMI of 30 kg·m^− 2^ or more. About 13.0% were in the highest quartile of county-level poverty, and 38.1% resided in the Southeast region. On average, participants reported 9.5 h/day of sedentary time, followed by 8.1 h/day in bed/sleeping and 6.4 h/day in active time. Participants provided recall data across all days of the week (Supplemental Table 1). Among those with one recall, the highest proportions were completed on Wednesday (18.5%) and Sunday (17.9%), and the lowest on Tuesday (8.6%). For participants with two recalls, the highest proportions were also on Sunday (22.8%) and Wednesday (15.7%), with the lowest on Thursday (9.4%). The remaining days showed relatively similar distributions across both groups.

**Table 1:** Descriptive characteristics of the study participants

Characteristics	Overall (*n* = 2,637)	Male (*n* = 1,458)	Female (*n* = 1,179)
Age in years	45.1 ± 0.51	46.2 ± 0.65	44.1 ± 0.70
Racial and/or ethnic groups			
Black, non-Hispanic	283 (11.0)	104 (8.8)	179 (13.0)
White, non-Hispanic	1,793 (63.6)	1,047 (66.2)	746 (61.0)
Hispanic	336 (17.0)	148 (14.2)	188 (19.7)
Asian	105 (3.6)	82 (5.0)	23 (2.2)
Other#	120 (4.9)	77 (5.8)	43 (4.1)
Marital status			
Married	1,431 (51.5)	857 (54.5)	574 (48.6)
Unmarried	1,206 (48.5)	601 (45.5)	605 (51.4)
Educational attainment			
High school or less	381 (34.6)	209 (35.5)	172 (33.6)
Some college/associate degree	1,019 (29.0)	523 (27.4)	496 (30.5)
Bachelor’s degree	721 (20.9)	424 (22.0)	297 (19.9)
Graduate degree	516 (15.5)	302 (15.1)	214 (16.0)
Household income ($)			
< 50,000	958 (41.2)	448 (37.4)	510 (45.0)
50,000–99,000	948 (34.1)	554 (36.5)	394 (31.8)
100,000–149,000	449 (15.5)	269 (15.6)	180 (15.4)
150,000+	282 (9.2)	187 (10.6)	95 (7.8)
Occupational status			
Working for pay	1,931 (67.2)	1,111 (70.2)	820 (64.2)
Not working—looking/laid off	130 (6.2)	63 (6.0)	67 (6.5)
Not working—other	173 (7.5)	42 (3.0)	131 (11.9)
Retired	287 (13.3)	173 (14.2)	114 (12.5)
Disabled	116 (5.8)	69 (6.6)	47 (4.9)
ACT24 recall durations (h·d^− 1^)*			
In bed/sleep time	8.1 ± 0.05	8.0 ± 0.09	8.3 ± 0.08
Sedentary time	9.5 ± 0.10	9.9 ± 0.13	9.1 ± 0.14
Active time	6.4 ± 0.09	6.1 ± 0.12	6.6 ± 0.13
Body mass index (kg·m^− 2^)	29.3 ± 0.18	29.1 ± 0.24	29.5 ± 0.29
< 25.0	774 (28.7)	395 (26.5)	379 (30.8)
25.0–29.9	885 (33.9)	548 (38.2)	337 (29.8)
≥ 30.0	978 (37.4)	515 (35.3)	463 (39.4)
County-level poverty †			
Quartile 1	769 (27.8)	433 (29.0)	336 (26.7)
Quartile 2	780 (30.0)	458 (32.5)	322 (27.6)
Quartile 3	797 (29.6)	411 (25.8)	386 (33.3)
Quartile 4	291 (12.6)	156 (12.7)	135 (12.4)
Region			
Northeast	383 (16.8)	396 (18.4)	166 (15.3)
Midwest	736 (20.6)	396 (20.0)	340 (21.2)
Southeast	857 (38.1)	471 (36.9)	386 (39.2)
West	661 (24.5)	374 (24.6)	287 (24.3)

Note: Categorical variables are presented as frequency (weighted %) and continuous variables as weighted means (SEs)

*ACT24 recall duration: sum of the total duration of individual physical activity behaviors reported on the previous day with at least one valid recall (4,161 recalls)

†Higher quartiles indicate higher levels of poverty.

# Other racial and/or ethnic groups include non-Hispanic adults reporting other or two or more racial and/or ethnic groups.

Table 2 shows the distribution of time spent in SB by life domains and subgroups. Males reported more (9.9 h/day) sedentary time than females (9.1 h/day). Asian adults reported the most (10.5 h/day) sedentary time, and Hispanic adults reported the least (8.9 h/day). Most of the sedentary time was accumulated in leisure activities, followed by work and transportation life domain activities.

**Table 2:** Time spent in sedentary behaviors (hours/day) by overall and life domains and subgroups, n recall = 4,161

Subgroups	Total	Life Domains					
		Leisure	Personal	Work	Household	Transport	Other
Overall	9.53 (9.33, 9.73)	4.34 (4.18, 4.51)	0.91 (0.86, 0.96)	1.87 (1.70, 2.04)	0.52 (0.47, 0.56)	1.09 (1.03, 1.15)	0.80 (0.68, 0.93)
Sex							
Male	9.93 (9.66, 10.20)	4.64 (4.42, 4.85)	0.94 (0.88, 1.01)	2.00 (1.76, 2.23)	0.38 (0.33, 0.44)	1.17 (1.06, 1.28)	0.80 (0.67, 0.93)
Female	9.14 (8.85, 9.43)	4.05 (3.86, 4.25)	0.87 (0.82, 0.93)	1.75 (1.51, 1.98)	0.65 (0.58, 0.71)	1.01 (0.95, 1.07)	0.81 (0.60, 1.01)
Racial and/or ethnic groups							
Black, non-Hispanic	9.80 (9.22, 10.38)	4.01 (3.53, 4.50)	0.79 (0.66, 0.92)	2.11 (1.69, 2.52)	0.41 (0.28, 0.55)	1.21 (0.94, 1.48)	1.26 (0.95, 1.57)
White, non-Hispanic	9.64 (9.42, 9.86)	4.72 (4.51, 4.92)	0.94 (0.89, 0.99)	1.82 (1.62, 2.01)	0.51 (0.44, 0.57)	1.08 (1.00, 1.15)	0.58 (0.49, 0.68)
Hispanic	8.93 (8.37, 9.48)	3.44 (3.07, 3.80)	0.83 (0.70, 0.97)	1.58 (1.23, 1.93)	0.65 (0.52, 0.78)	0.99 (0.87, 1.12)	1.43 (0.98, 1.89)
Asian	10.55 (9.44, 11.65)	3.80 (3.12, 4.47)	1.20 (0.83, 1.57)	3.11 (2.34, 3.88)	0.40 (0.20, 0.61)	1.31 (0.79, 1.82)	0.74 (0.54, 0.93)
Other*	8.85 (7.90, 9.81)	3.74 (3.26, 4.23)	0.79 (0.68, 0.90)	2.10 (1.29, 2.90)	0.49 (0.19, 0.79)	1.15 (0.91, 1.39)	0.58 (0.37, 0.80)

Note: Variables are presented as weighted means (95% confidence intervals).

* Other racial and/or ethnic groups include non-Hispanic adults reporting other or two or more racial and/or ethnic groups.

### Association between race and/or ethnicity specific residential segregation and sedentary time

Table 3 shows the association between race and/or ethnicity specific residential segregation and time spent (hours/day) in SB. NH Black segregation was not related to sedentary time (β = -0.30 [-2.53, 1.94]), and Hispanic segregation also showed no relationship (β = 0.32, [-1.64, 2.28]). We observed similar findings when NH White adults were used as the reference group in the isolation index (Supplemental Table 2).

**Table 3:** Race and/or ethnicity specific association between residential segregation and time spent (hours/day) in sedentary behaviors in US adults

Isolation Index by Racial and/or Ethnic Groups	Model 1		Model 2	
	β (95% CI)	*p*	β (95% CI)	*p*
Non-Hispanic Black (vs. All Other), n recall = 426	-0.64 (-2.65, 1.36)	0.520	-0.30 (-2.53, 1.94)	0.790
Hispanic (vs. All Other), n recall = 508	0.04 (-2.66, 2.75)	0.975	0.32 (-1.64, 2.28)	0.743

Model 1 was adjusted for age and sex.

Model 2 was adjusted for variables in model 1 plus marital status, educational attainment, occupation, body mass index category, county-level poverty, and census regions.

### Association between Race and/or ethnicity specific residential segregation and sedentary time stratified by sex

In the sex-stratified analysis (Table 4), we found no significant associations between county-level residential segregation and sedentary time among NH Black males and females, and Hispanic males and females. Likewise, there were no significant associations when using NH White adults as the reference group in isolation index calculations (Supplemental Table 3).

**Table 4:** Race and/or ethnicity specific association between residential segregation and time spent (hours/day) in sedentary behaviors in US adults, stratified by sex

	Male					Female			
Isolation Index by Racial and/ or Ethnic Groups	Model 1		Model 2			Model 1		Model 2	
	β (95% CI)	*p*	β (95% CI)	*p*		β (95% CI)	*p*	β (95% CI)	*p*
Non-Hispanic Black (vs. All Other), n recall for male = 157, n recall for female = 269	0.15 (-3.70, 3.99)	0.939	1.07 (-3.38, 5.51)	0.628		-0.89 (-3.42, 1.65)	0.483	-1.45 (-4.48, 1.58)	0.338
Hispanic (vs. All Other), n recall for male = 231, n recall for female = 277	-0.69 (-3.78, 2.41)	0.656	-0.64 (-4.30, 3.02)	0.724		0.69 (-2.97, 4.35)	0.705	0.95 (-2.11, 4.02)	0.533

Model 1 was adjusted for age.

Model 2 was adjusted for variables in model 1 plus marital status, educational attainment, occupation, body mass index category, county-level poverty, and census regions.

### Associations between race and/or ethnicity specific residential segregation and domain-specific sedentary time

Among Hispanic adults, residential segregation was marginally and positively associated with sedentary time, but only in the personal domain (β = 0.65, [0.00, 1.29]). For NH Black and Hispanic adults, other associations with domain-specific sedentary time, including leisure, personal, work, household, transport, and other domains, did not reach statistical significance (Table 5).

**Table 5:** Race and/or ethnicity specific association between residential segregation and time spent (hours/day) in sedentary behaviors in US adults by life domains

	Leisure		Personal		Work		Household		Transport		Other	
Isolation Index by Racial and/or Ethnic Groups	Model 1	Model 2	Model 1	Model 2	Model 1	Model 2	Model 1	Model 2	Model 1	Model 2	Model 1	Model 2
	β (95% CI)	β (95% CI)	β (95% CI)	β (95% CI)	β (95% CI)	β (95% CI)	β (95% CI)	β (95% CI)	β (95% CI)	β (95% CI)	β (95% CI)	β (95% CI)
Non-Hispanic Black (vs. All Other), n recall = 426	1.02 (-0.93, 2.97)	0.30 (-1.81, 2.41)	0.04 (-0.47, 0.55)	-0.06 (-0.57, 0.45)	-2.00 (-4.12, 0.12)	0.03 (-2.02, 2.09)	0.28 (-0.33, 0.88)	0.36 (-0.55, 1.27)	-0.58 (-1.42, 0.26)	-0.99 (-2.30, 0.33)	0.60 (-1.11, 2.32)	0.06 (-1.88, 2.00)
Hispanic (vs. All Other), n recall = 508	0.48 (-1.22, 2.17)	0.18 (-1.59, 1.94)	0.56 (-0.09, 1.22)	0.65 (0.00, 1.29)	-0.61 (-2.00, 0.78)	-0.48 (-1.80, 0.84)	-0.07 (-0.75, 0.62)	-0.17 (-0.98, 0.64)	0.02 (-0.51, 0.56)	0.24 (-0.40, 0.88)	-0.34 (-3.52, 2.83)	-0.09 (-1.81, 1.63)

Model 1 was adjusted for age and sex.

Model 2 was adjusted for variables in model 1 plus marital status, educational attainment, occupation, household income, body mass index category, county-level poverty, and census regions

The boldface indicates a significant association at *p* < 0.05

## Discussion

Evidence for the association between residential segregation and time engaged in SB is highly limited. To overcome this gap, we examined the relationships between residential segregation and sedentary time among NH Black and Hispanic adults in this nationwide sample of US adults. In our cross-sectional analyses, living in more segregated counties was not associated with the time spent in SB among NH Black and Hispanic adults, both in overall and sex-stratified analyses. In domain-specific analyses, Hispanic adults living in segregated counties showed a marginally positive association with sedentary time in personal life domains only.

This study is among the first to examine associations between residential segregation and detailed population-based estimates of SB of US adults, encompassing all major life domains, using a novel validated ACT24 tool. Thus, it is difficult to compare all our results with other studies. However, since some studies used other forms of sedentary or physical activity behavior, it was reasonable to compare our results with their findings. We found no statistically significant association between residential segregation and sedentary time among NH Black adults. This finding is consistent with a recent CARDIA study, showing non-significant cross-sectional and longitudinal relationships between residential segregation assessed using Getis-Ord Local Statistic (known as Gi* statistic) and accelerometer-measured total sedentary time among Black adults (vs. White counterparts) [33].

Few studies have examined relationships between residential segregation and other forms of physical activity behavior. For instance, a study using the 2001 Behavioral Risk Factor Surveillance System (BRFSS) indicated that residential segregation (as measured by dissimilarity index) was not associated with physical inactivity in Black adults-only analysis [25]. In another 2000 BRFSS study, residential segregation at the metropolitan statistical area (MSA) level, assessed by the isolation index, was not associated with any exercise (yes/no) in the past month among Black adults [44]. In contrast, a study from North Carolina reported a significant positive association between the percent of Black adults living in segregated neighborhoods and higher odds of engaging in more minutes of walking and moderate to vigorous physical activity per week, as compared to those living in predominantly European American neighborhoods [45].

These findings regarding the association between residential segregation and sedentary or physical activity behaviors are mixed and inconclusive. Most existing studies found no significant relationship between residential segregation (measured using various indices) and these behaviors across different study samples. Thus, the existing evidence suggests that residential segregation alone may not have a significant effect on SB. The reasons for the non-significant associations are not fully understood. Different occupations may influence the amount of time spent in physical activity behavior [46]. A relatively small sample size (*n* = 283 for NH Black adults and *n* = 336 for Hispanic adults) in our study could be one reason for our failure to reach statistical significance. Still, this explanation does not hold for other studies with larger sample sizes (*n* = 2,120 for CARDIA study, and 11,114 for BRFSS study) [33, 44]. In our study, residential segregation was assessed at the county level; thus, the participants’ perceptions and experiences regarding how their neighborhoods facilitate or inhibit their SB remained unmeasured/unknown. Residential segregation is a socially constructed phenomenon [47]. Thus, people’s perspectives and experiences about their neighborhoods are essential to understanding their role in SB. In a mixed-methods study, qualitative interviews revealed some factors that may relate to one’s activity behaviors [45]. These included the availability, proximity, and usage of physical activity-promoting facilities, the age of the neighbors, familiarity with neighbors, feeling of safety, and social cohesion [45]. Other unmeasured factors, such as changes in neighborhood characteristics over time and length of stay in neighborhoods, are also important to consider [33]. Further research is warranted to explore neighborhood-level factors in order to more comprehensively understand the lack of a significant association between residential segregation and SB.

Our results focusing on Hispanic adults indicated no association between residential segregation and sedentary time. This finding is different from the only other national study of Hispanic adults, indicating that living in segregated MSAs was significantly associated with an 18% lower likelihood of exercising compared to those living in less segregated MSAs [48]. However, a direct comparison between these two studies may not be possible because of methodological variations in measuring segregation and assessing physical activity. For instance, we used the county-level isolation index, whereas the study on Hispanic adults used the dissimilarity index at MSAs. Additionally, we quantified SB using a comprehensive ACT24 tool, which consists of a list of over 170 individual activities organized in 14 major categories, in contrast to the other study’s use of a single-item question (yes/no) on any exercise in the past month. Furthermore, less exercise does not imply one being more sedentary.

This is the first nationwide study of US adults to investigate the relationship between residential segregation and a comprehensive population-based estimate of SB, including an exploration of all major life domains, which is the strength of this study. However, there are some limitations. First, subjective measures of SB could lead to social desirability bias, which could further lead to an under-reporting of SB [17]. However, one study tested this hypothesis using a previous day recall (compared with an accelerometer measure) and found no evidence of social desirability bias in reported SB [49]. Another previous study showed that the ACT24 method accurately estimated sedentary time at the population level compared to accelerometer-derived measures [38]. Second, there might be seasonal variation in SB. Given the larger differences in physical activity between summer and winter [50, 51], the current study chose to collect data in the fall to minimize the extremes of season variation and more accurately estimate SB in the population. Third, the cross-sectional nature of this study limited its ability to establish a causal relationship between segregation and SB. Finally, the small sample size of NH Black and Hispanic adults might have led to lower statistical power to detect significant relationships between segregation and SB. Additional research with larger samples of minority groups is needed to ensure greater representativeness and yield more robust results.

## Conclusions

In this nationwide sample of US adults, we found no significant relationships between county-level residential segregation and SB among NH Black and Hispanic adults. Additionally, we did not find statistically significant moderation effects by sex. Given the scarce and inconsistent evidence on this topic, future studies could aim to replicate this line of research with larger samples of underrepresented minority populations and further explore sex- and life domain-specific variation in the association between residential segregation and SB. Additionally, more work is needed to investigate how neighborhood conditions (e.g., built and social environments) are related to SB.

## Supplementary Information

Below is the link to the electronic supplementary material.

Supplementary Material 1

## Data Availability

The data described in the study are available from the National Cancer Institute (NCI), but restrictions apply to their availability. However, the data can be obtained from the corresponding author upon reasonable request and with the permission of the NCI.

## Declarations

### Disclaimer

This research was supported by the Intramural Research Program of the NIH. The contributions of the NIH authors were made as part of their official duties as NIH federal employees, are in compliance with agency policy requirements, and are considered Works of the United States Government. However, the findings and conclusions presented in this paper are those of the authors and do not necessarily reflect the views of the NIMHD, NCI, NIH, or the U.S. Department of Health and Human Services.

## Abbreviations

ACT24: Activities Completed over Time in 24-hours
BRFSS: Behavioral Risk Factor Surveillance System
CARDIA: Coronary Artery Risk Development in Young Adults
CI: confidence interval
MET: metabolic equivalent
MSA: metropolitan statistical area
NH: non-Hispanic
NORC: National Opinion Research Center
SB: sedentary behavior
