# Supplementary material for: County-level residential segregation and sedentary behavior in US adults
Source: J Act Sedentary Sleep Behav. 2025 Sep 2;4:15. doi: 10.1186/s44167-025-00084-w (PMC12403554; doi:10.1186/s44167-025-00084-w)
Supplement: Supplementary file 1 — Supplementary Material 1 [file 44167_2025_84_MOESM1_ESM.docx]

**Supplemental Material**

**County-Level Residential Segregation and Sedentary Behavior in US Adults**

**Journal:** *Journal of Activity, Sedentary and Sleep Behaviors*

| Supplemental Table 1. Frequency of one and two recalls by day of the week | | |
| --- | --- | --- |
| **Day of the week** | **One recall = 1,113, n (%)** | **Two recalls = 3,048, n (%)** |
| Monday | 157 (14.1) | 404 (13.3) |
| Tuesday | 96 (8.6) | 366 (12.0) |
| Wednesday | 206 (18.5) | 478 (15.7) |
| Thursday | 163 (14.7) | 288 (9.4) |
| Friday | 144 (12.9) | 414 (13.6) |
| Saturday | 148 (13.3) | 404 (13.2) |
| Sunday | 199 (17.9) | 694 (22.8) |

| Supplemental Table 2. Race and/or ethnicity specific association between residential segregation and time spent (hours/day) in sedentary behaviors in US Adults | | | | |
| --- | --- | --- | --- | --- |
| **Isolation Index by Racial and/or Ethnic Groups** | **Model 1** | | **Model 2** | |
|  | **β (95% CI)** | **p-value** | **β (95% CI)** | **p-value** |
| Non-Hispanic Black (vs. White) | -0.56 (-2.61, 1.48) | 0.581 | -0.28 (-2.28, 1.73) | 0.783 |
| Hispanic (vs. White) | 0.50 (-2.04, 3.04) | 0.693 | 0.84 (-1.04, 2.72) | 0.370 |
| Model 1 was adjusted for age and sex. | | | | |
| Model 2 was adjusted for variables in model 1 plus marital status, educational attainment, occupation, body mass index category, county-level poverty, and census regions. | | | | |

| Supplemental Table 3. Race and/or ethnicity specific association between residential segregation and time spent (hours/day) in sedentary behaviors in US Adults, stratified by sex | | | | | | | | | |
| --- | --- | --- | --- | --- | --- | --- | --- | --- | --- |
|  | **Male** | | | |  | **Female** | | | |
| **Isolation Index by Racial and/or Ethnic Groups** | **Model 1** | | **Model 2** | |  | **Model 1** | | **Model 2** | |
|  | **β (95% CI)** | **p-value** | **β (95% CI)** | **p-value** |  | **β (95% CI)** | **p-value** | **β (95% CI)** | **p-value** |
| Non-Hispanic Black (vs. White) | 0.76 (-3.42, 4.95) | 0.713 | 1.41 (-2.85, 5.67) | 0.505 |  | -1.13 (-3.54, 1.28) | 0.348 | -1.36 (-3.92, 1.21) | 0.292 |
| Hispanic (vs. White) | 0.61 (-2.35, 3.56) | 0.682 | 0.88 (-2.44, 4.20) | 0.595 |  | 0.66 (-2.92, 4.25) | 0.711 | 0.90 (-1.90, 3.70) | 0.520 |
| Model 1 was adjusted for age. | | | | | | | | | |
| Model 2 was adjusted for variables in model 1 plus marital status, educational attainment, occupation, body mass index category, county-level poverty, and census regions. | | | | | | | | | |
